# Perceptions of Nurse Practitioners and Physician Assistants/Associates Toward the Concept of Developing an Advanced Practice Postgraduate Residency/Fellowship Program at a Large Academic Medical Center

**DOI:** 10.7759/cureus.67820

**Published:** 2024-08-26

**Authors:** Vasco Deon Kidd, Geraldina Douglas

**Affiliations:** 1 Orthopedic Surgery, University of California Irvine School of Medicine, Irvine, USA; 2 Perioperative Services, University of California Irvine School of Medicine, Irvine, USA

**Keywords:** academic medical center, healthcare access, transition-to-practice, postgraduate education, fellowship, residency, advanced practice providers, nurse practitioner, physician assistant/associate

## Abstract

Introduction

There have been exponential growth and increased interest in postgraduate residency/fellowship formalized training among nurse practitioners (NPs) and physician assistants/associates (PAs). Although not a requirement for state licensure and entry-level practice, postgraduate NP and PA residency/fellowship programs offer a structured pathway for new graduates and experienced PAs and NPs looking to transition into a new medical or surgical specialty/subspecialty. In this article, we examine the perceptions of employed NPs and PAs toward postgraduate training including the concept of developing a program at our institution.

Methodology

This was a cross-sectional single-large academic medical center (AMC) study, where an anonymous electronic survey was initially developed by the director of advanced practice providers (APPs). The survey was piloted by members of the Advanced Practice Council (APC) comprising NPs, PAs, and certified registered nurse anesthetists (CRNAs), and their collective feedback was used to finalize the survey prior to distribution. Descriptive statistics were used to describe and summarize the data. In addition, we assessed the association between respondents' characteristics and perceptions regarding NP and PA residency/fellowship programs using chi-squared tests of independence.

Results

The majority of the respondents (69.1%; 65/94) believed that starting an advanced practice residency/fellowship program at our AMC has many benefits such as increasing specialty-specific knowledge and training (79.8%; 75/94), improving medical decision-making (73.4%; 69/94), promoting professional development and clinical education (73.4%; 69/94), and improving procedural competency (60.6%; 57/94). Moreover, over half of the respondents (53.2%; 50/94) indicated that there is value in an APP residency/fellowship program obtaining optional accreditation. Additionally, over half of the respondents (56.4%; 53/94) indicated that they would be interested in participating as a clinical preceptor if an APP residency/fellowship program was developed at our AMC. Lastly, about one-third of the respondents felt strongly that APP residency/fellowship training programs should offer post-professional doctoral degree options such as Doctor of Nursing Practice (DNP), Doctor of Medical Science (DMSc), and Doctor of Health Science (DHSc).

Conclusion

Although most respondents have never completed a formalized postgraduate training program and were less familiar with the published literature regarding these programs, our findings suggest that the attitudes of employed NPs and PAs are generally positive toward the concept of postgraduate specialty-specific training.

## Introduction

The exponential growth of advanced practice providers (APPs) also referred to as nurse practitioners (NPs) and physician assistants/associates (PAs) represents a large component of the healthcare workforce. According to the Bureau of Labor Statistics (BLS), employment of NPs is projected to increase by 45% from 2022 to 2032 and for PAs by 26.5% during the same period [1,2]. There are numerous factors contributing to the demand of APPs such as an increasing aging population and an aging physician workforce nearing retirement [3]. Although NPs and PAs complete graduate-level education, the training, certification, and licensure requirements differ between professions. For example, PAs are educated as generalists in the medical model, and NPs are educated in at least one of six population foci (family/individuals across lifespan, women's health, adult/gerontology, pediatrics, psychiatric/mental health, and neonatal) [4]. 

The research has elucidated several distinct advantages of employing APPs such as increasing physician productivity, improving patient safety and outcomes, and bolstering healthcare system capacity through interprofessional collaboration [5-10]. In addition, APPs are seeing a greater number of patients, who are sicker and require more complex care [9]. For newly minted APPs entering the workforce or those looking to change specialties, some may require additional support to help them navigate this career transition. Prior research demonstrates that novice NPs have expressed interest in formalized postgraduate program training due to perceptions about readiness to enter clinic practice [11-13]. APP formalized training programs range from 12 to 24 months in length and are designed to help facilitate transition into practice by enhancing specialty-specific knowledge, procedural skills, and confidence level. 

While PA postgraduate formalized training has been around for over 50 years and NP postgraduate training for 17 years, there is insufficient data on the direct impact of NP and PA postgraduate training on patient outcomes. It is not surprising that postgraduate training has engendered excitement in some APPs and skepticism in others. Therefore, the purpose of this study is to examine the attitudes of NP and PA career staff toward formalized APP postgraduate training at a large academic medical center (AMC). Moreover, we will determine if there is an association between respondents' characteristics such as clinical profession, clinical area worked, and years worked at the organization and respondents' perceptions regarding the value in obtaining optional accreditation, participating as a clinical preceptor, and post-professional doctoral degree options.

## Materials and methods

An anonymous electronic survey was developed by the director of APPs. The survey was piloted by members of the Advanced Practice Council (APC) comprising NPs, PAs, and certified registered nurse anesthetists (CRNAs), and their collective feedback was used to finalize the survey prior to distribution. The survey was built using the Qualtrics survey platform (Qualtrics Inc., Provo, UT, USA). Participants received an email introduction to the survey containing all the necessary elements of written consent, and submission of the survey indicated the respondents' consent to participate. CRNAs were excluded from participating in this study as CRNA postgraduate fellowship training is not well described in the literature. However, NPs and PAs were eligible to participate in this study, including those who were doctorally prepared and/or had completed an APP residency/fellowship program. Confidentiality was maintained throughout the study. Using an online sample size calculator, we estimated that 126 (68%) or more survey responses were needed to have a confidence level of 95% within a 5% margin of error. The study period was between July and August 2023. Multiple email reminders were sent out during the study period to improve participant engagement. The Institutional Review Board of the University of California, Irvine (UCI), approved the study (approval number: 3619). 

Analysis

Data were imported into and analyzed using IBM SPSS Statistics for Windows, V. 23.0 (IBM Corp., Armonk, NY, USA). Frequency tables were used to summarize the data. To determine the association between respondents' characteristics and perceptions regarding the APP residency/fellowship program, chi-squared tests of independence were performed [14]. The chi-squared test of independence is a statistical hypothesis test used to determine whether two categorical variables are related [8]. When the expected value of a cell in the contingency table was less than 5, the p-value of the chi-squared test was computed by the Monte Carlo method [15,16]. For any tests, a p-value less than 0.05 indicated statistical significance.

## Results

The overall response rate was 50.8% (94/185). Nineteen respondents skipped more than half of the survey questions and were excluded from the data analysis. Table 1 summarizes the survey responses. Nearly three-fourths of the respondents 72.3% (68/94) were NPs. Around two-thirds of the respondents (66.0%; 62/94) worked in medical specialties. More than half of the respondents have been working for UCI for over five years. Around 69.1% (65/94) felt there is value in starting a postgraduate APP residency/fellowship at UCI. Around two-thirds of the respondents believed that both onboarding programs and APP residency/fellowship programs provide greater organizational value. 

**Table 1:** Summary of survey responses Q6: Question 6 was a multi-select question and the % of each response was computed by (frequency/94)*100. APP: advanced practice provider; PA: physician assistant/associate; NP: nurse practitioner; UCI: University of California, Irvine; DNP: Doctor of Nursing Practice; DMSc: Doctor of Medical Science; DHSc: Doctor of Health Science

Survey questions	N (%)
Q1. Are you a	
NP	68 (72.3)
PA	26 (27.7)
Q2. Do you work in the following areas?	
Medical specialty	62 (66.0)
Surgical specialty	31 (33.0)
Missing response	1 (1.1)
Q3. How long have you been working for UCI?	
<1 year	7 (7.4)
1-2 years	15 (16.0)
3-5 years	19 (20.2)
>5 years	53 (56.4)
Q4. Do you feel there is value in starting a postgraduate APP residency/fellowship at UCI?	
No	6 (6.4)
Unsure	23 (24.5)
Yes	65 (69.1)
Q5. Which of the following statements adequately reflects your opinion about onboarding versus APP residency/fellowship programs?	
I believe there is greater organizational value in developing onboarding programs (typically, six months or less) for employees as opposed to starting a formalized residency/fellowship program	11 (11.7)
I believe there is greater organizational value in developing residency and fellowship programs that award a certificate of completion	16 (17.0)
I believe that both onboarding programs and APP residency/fellowship programs provide greater organizational value	62 (66.0)
Missing response	5 (5.3)
Q6. What is the perceived value of starting an APP residency/fellowship program at UCI?	
Improves recruitment and retention of APPs	53 (56.4)
Improves job satisfaction of new employees	45 (47.9)
Improves medical decision-making	69 (73.4)
Improves procedural competency	57 (60.6)
Increases specialty-specific knowledge and training	75 (79.8)
Facilitates role transition and improves onboarding into a new specialty	55 (58.5)
Promotes staff engagement	32 (34.0)
Promotes professional development and clinical education	69 (73.4)
Improves specialty-specific billing/coding	27 (28.7)
Supports internal transfer opportunities for APPs	40 (42.6)
Other	4 (4.3)
Q7. Have you previously completed a postgraduate APP residency/fellowship training program?	
No	83 (88.3)
Yes	6 (6.4)
Missing response	5 (5.3)
Q8. Do you think there is value in an APP residency/fellowship program obtaining optional accreditation?	
No	13 (13.8)
Unsure	26 (27.7)
Yes	50 (53.2)
Missing response	5 (5.3)
Q9. If an APP residency/fellowship program was developed at UCI, would you be interested in participating as a clinical preceptor?	
No	11 (11.7)
Unsure	25 (26.6)
Yes	53 (56.4)
Missing response	5 (5.3)
Q10. Are you familiar with the current published literature regarding APP residency/fellowship programs in the United States?	
No	58 (61.7)
Somewhat	17 (18.1)
Yes	13 (13.8)
Missing response	6 (6.4)
Q11. Do you feel strongly that APP residency/fellowship training programs should offer post-professional doctoral degree options such as DNP, DMSc, and DHSc?	
No	21 (22.3)
Unsure	36 (38.3)
Yes	30 (31.9)
Missing response	7 (7.4)
Q12. Do you believe that developing APP residency/fellowship programs within the organization would improve patient access and provider retention?	
No	10 (10.6)
Unsure	17 (18.1)
Yes	60 (63.8)
Missing response	7 (7.4)

The majority of the respondents believed that starting an APP residency/fellowship program at UCI would increase specialty-specific knowledge and training (79.8%; 75/94), improve medical decision-making (73.4%; 69/94), and promote professional development and clinical education (73.4%; 69/94). Only six (6.4%) of the respondents have previously completed a postgraduate APP residency/fellowship training program. Over half of the respondents think that there is value in an APP residency/fellowship program obtaining optional accreditation. Moreover, 56.4% (53/94) of the respondents indicated that they would be interested in participating as a clinical preceptor if an APP residency/fellowship program was developed at UCI. Further, 32% (30/94) of the respondents were familiar or somewhat familiar with the current published literature regarding APP residency/fellowship programs in the United States. 

Tables 2-4 show the cross-tabulation between respondents' characteristics, such as clinical profession (Q1), clinical area worked (Q2), and years worked at the organization (Q3), and respondents' perceptions regarding value in an APP residency/fellowship program obtaining optional accreditation (Q8), participating as a clinical preceptor if an APP residency/fellowship program was developed at UCI (Q9), and an APP residency/fellowship program offering post-professional doctoral degree options (Q11). Note that for the purpose of the analysis, data of years worked at the organization were regrouped into two groups: 0-5 years vs. >5 years.

**Table 2:** Cross-tabulation N (%) between Q1 (clinical profession) and Q8, Q9, and Q11 *indicates statistical significance at the 0.05 level. χ^2^: chi-squared test statistic; df: degrees of freedom; p: p-value; Q1: survey question 1; Q8: survey question 8; Q9: survey question 9; Q11: survey question 11; N: sample size; NP: nurse practitioner; PA: physician assistant/associate

Survey questions					
(N)	NP	PA	χ^2^	Df	P
Q8 (N = 65 NPs and N = 24 PAs)					
No	7 (10.8)	6 (25.0)	3.056	2	0.258
Unsure	19 (29.2)	7 (29.2)	-	-	-
Yes	39 (60.0)	11 (45.8)	-	-	-
Q9 (N = 65 NPs and N = 24 PAs)					
No	6 (9.2)	5 (20.8)	4.710	2	0.122
Unsure	16 (24.6)	9 (37.5)	-	-	-
Yes	43 (66.2)	10 (41.7)	-	-	-
Q11 (N = 65 NPs and N = 22 PAs)					
No	15 (23.1)	6 (27.3)	0.682	2	0.711
Unsure	26 (40.0)	10 (45.5)	-	-	-
Yes	24 (36.9)	6 (27.3)	-	-	-

**Table 3:** Association between Q2 (clinical area worked) and Q8, Q9, and Q11 *indicates statistical significance at the 0.05 level. χ^2^: chi-squared test statistic; df: degrees of freedom; P: p-value; Q2: survey question 2; Q8: survey question 8; Q9: survey question 9; Q11: survey question 11; N: sample size

Survey questions						
(N)	Medical specialty	Surgical specialty	χ^2^	df		P
Q8 (N = 88)						
No	6 (10.2)	6 (20.7)	6.344	2	0.044*	
Unsure	14 (23.7)	12 (41.4)	-	-	-	
Yes	39 (66.1)	11 (37.9)	-	-	-	
Q9 (N = 88)						
No	4 (6.8)	6 (20.7)	4.441	2	0.092	
Unsure	16 (27.1)	9 (31.0)	-	-	-	
Yes	39 (66.1)	14 (48.3)	-	-		
Q11 (N = 86)						
No	11 (19.0)	9 (32.1)	1.950	2	0.377	
Unsure	25 (43.1)	11 (39.3)	-	-	-	
Yes	22 (37.9)	8 (28.6)	-	-	-	

**Table 4:** Association between Q3 (years worked at the organization) and Q8, Q9, and Q11 *indicates statistical significance at the 0.05 level. χ^2^: chi-squared test statistic; df: degrees of freedom; p: p-value; Q3: survey question 3; Q8: survey question 8; Q9: survey question 9; Q11: survey question 11; N: sample size

Survey questions					
	0-5 years	>5 years	χ^2^	df	P
Q8 (N = 38 and N = 51)					
No	5 (13.2)	8 (15.7)	0.509	2	0.775
Unsure	10 (26.3)	16 (31.4)	-	-	-
Yes	23 (60.5)	27 (52.9)	-	-	-
Q9 (N = 38 and N = 51)					
No	3 (7.9)	8 (15.7)	1.368	2	0.539
Unsure	12 (31.6)	13 (25.5)	-	-	-
Yes	23 (60.5)	30 (58.8)	-	-	-
Q11 (N = 38 and N = 49)					
No	7 (18.4)	14 (28.6)	1.410	2	0.494
Unsure	16 (42.1)	20 (40.8)	-	-	-
Yes	15 (39.5)	15 (30.6)	-	-	-

There was no association between clinical profession (Q1) and respondents' perceptions regarding value in an APP residency/fellowship program obtaining optional accreditation (Q8: χ^2^(2, N = 89) = 3.056, p = 0.258), participating as a clinical preceptor if an APP residency/fellowship program was developed at UCI (Q9: χ^2^(2, N = 89) = 4.710, p = 0.122), and an APP residency/fellowship program offering post-professional doctoral degree options (Q11: χ^2^(2, N = 87) = 0.682, p = 0.711) (Table 2).

There was a statistically significant association between clinical area worked (Q2) and respondents' perceptions regarding value in an APP residency/fellowship program obtaining optional accreditation (Q8: χ^2^(2, N = 89) = 6.344, p = 0.044) (Table 3). The majority of the respondents (66.1%) working in a medical specialty believed that there was value in an APP residency/fellowship program obtaining optional accreditation, while only less than 40% of the respondents (37.9%) working in a surgical specialty concurred. There was no association between clinical area worked (Q2) and respondents' perceptions regarding participating as a clinical preceptor if an APP residency/fellowship program was developed at UCI (Q9: χ^2^(2, N = 88) = 4.441, p = 0.092) and an APP residency/fellowship program offering post-professional doctoral degree options (Q11: χ^2^(2, N = 86) = 1.950, p = 0.377) (Table 3).

There was no association between years worked at the organization (Q3) and respondents' perceptions regarding value in an APP residency/fellowship program obtaining optional accreditation (Q8: χ^2^(2, N = 89) = 0.509, p = 0.775), participating as a clinical preceptor if an APP residency/fellowship program was developed at UCI (Q9: χ^2^(2, N = 89) = 1.368, p = 0.539), and an APP residency/fellowship program offering post-professional doctoral degree options (Q11: χ^2^(2, N = 87) = 1.410, p = 0.494) (Table 4).

## Discussion

This study contributes to the ongoing dialogue and evolving landscape of NP and PA postgraduate training. The aims of this study were to examine and summarize respondent survey data including determining if there is an association between respondents' characteristics and perceptions regarding the development of an APP residency/fellowship program. According to the data, most of the respondents (56.4%; 53/94) have been employed for over five years at our institution. Additionally, the study findings indicated that more than half of the respondents felt that starting an APP residency/fellowship program at our institution would increase specialty-specific knowledge and training, improve medical decision-making, and promote professional development and clinical education, including facilitating role transition. These findings correspond with prior research of postgraduate trainees [17,18]. Moreover, nearly two-thirds of the respondents (63.8%; 60/94) believed that developing APP residency/fellowship programs within the organization would improve patient access and provider retention. Less than half of the respondents felt that an APP residency program would improve the job satisfaction of new employees, promote staff engagement, and support internal transfer opportunities for APPs. 

Furthermore, most respondents felt that developing both onboarding programs (<6 months) and APP residency/fellowship program would provide greater organizational value. It is important to note that APP new employee onboarding programs vary by length, size, and scope of work versus APP postgraduate fellowships/residencies which offer 12-24 months of immersive clinical and didactic training at a reduced salary [19,20]. 

In terms of optional accreditation, although there has been an uptick in the number of accredited postgraduate APP programs in the United States, slightly more than half of respondents felt there was value in obtaining accreditation. Most respondents employed within a medical specialty favored obtaining accreditation, while slightly greater than a third of respondents employed within a surgical specialty saw value in obtaining accreditation. While there are multiple pathways for APP postgraduate programs to obtain voluntary accreditation, the value, effectiveness, and impact of external validation remain largely unanswered [21].

Regarding degree options, while very few postgraduate residencies/fellowships have partnered with academic institutions to offer post-professional doctorates, approximately one-third of the respondents felt strongly that APP residency/fellowship training programs should offer post-professional doctoral degree options such as Doctor of Nursing Practice (DNP), Doctor of Medical Science (DMSc), and Doctor of Health Science (DHSc) [22,23]. It is not surprising, as the direct impact of postgraduate trainees pursuing doctoral degrees during or following formalized specialty-specific training is largely unknown [24].

In summary, the data suggests that most respondents held favorable opinions about postgraduate programs including the notion of developing one at our AMC, although most respondents were unfamiliar with the current published literature regarding these formalized programs. Perhaps being employed at an AMC and exposure to resident physicians and fellows may have led to positive attitudes toward postgraduate training; however, this is speculative. 

Despite these interesting findings, our study has some limitations. First, this was a single-institution study with a small sample size; thus, our results may not be generalizable. Moreover, these findings represent the opinions of the NPs and PAs which may vary geographically and across the UCI ecosystem. Therefore, expanding this survey to other institutions using a mixed-method approach would improve the accuracy and generalizability of results. Furthermore, PAs were underrepresented in this study, partly due to the ratio of employed NPs to PAs within the institution where the study was undertaken. Consequently, the results may not fully represent the perceptions of all employed NPs and PAs within our AMC. 

## Conclusions

To our knowledge, this is the first study to examine the attitudes of both NPs and PAs from medical and surgical specialties regarding formalized postgraduate training. Our study findings indicate that among respondents, the majority had a positive attitude toward postgraduate training. Specifically, the research highlights that most respondents believe that there are numerous organizational benefits to developing both onboarding programs (six months or less) and formalized APP postgraduate residency/fellowship programs. However, there was no association detected between the clinical profession of the respondents and their perceptions regarding the value in an APP residency/fellowship program obtaining optional accreditation, participating as a clinical preceptor if an APP residency/fellowship program was developed at UCI, and an APP residency/fellowship program offering post-professional doctoral degree options. Additional research is warranted to better understand the factors that may contribute to attitudes and beliefs related to formalized NP and PA residency/fellowship training by all stakeholders.
